# Operationalising participatory action research to evaluate early years’ population health services

**DOI:** 10.1186/s12889-025-22183-8

**Published:** 2025-03-10

**Authors:** Katie Chadd, Mariam Malik, Anuj Kapilashrami

**Affiliations:** 1 School of Health & Social Care, University of Essex, Wivenhoe Park, Colchester, Essex CO4 3SQ UK; 2 Centre for Global Health & Intersectional Equity Research, Institute for Public Health & Well- being, Knowledge Gateway, University of Essex, Clingoe House, Wivenhoe Park, Colchester, Essex CO4 3ZL UK

**Keywords:** Early years, Participatory action research, Photovoice, Public health, Social determinants of health, Child development

## Abstract

**Background:**

Early years interventions are critical to children’s health and development and are emerging as core to public health programmes in the UK and globally. Evaluating such interventions is complex. The study reported in this article evaluates a place-based public health initiative ‘A Better Start Southend’ (ABSS) aimed at facilitating early years’ development specifically. It centres on examining the access barriers and facilitators experienced by parents and young children, as perceived by health professionals providing these services.

**Methods:**

This paper illustrates the utilisation of participatory action research (PAR) approach, employing creative methods, including spider grams, service mapping and photovoice, with health professionals delivering ABSS services. PAR methods enabled exploration of community resources that facilitate or impede early childhood development in the local context.

**Results:**

Operationalising PAR yielded critical providers’ perspectives on key challenges of delivering these programmes, and the factors that in their view impeded their uptake by families and hence effectiveness. The approach provided space for authentic knowledge production through critical reflexive enquiry, exchange, collaborative dialogue and transformation. Through the process, participants revealed the social and commercial determinants of childhood development and how these determined the reach and success of ABSS. Health professionals especially highlighted poor-quality housing, poor public transport, the cost-of-living crisis and harmful commercial marketing practices as key barriers to promoting good early childhood development. System-wide barriers were also reported and included poor resourcing of health and social care services, lack of culturally and linguistically accessible provisions, and exclusionary practices creating inequitable access to health for many families and children.

**Conclusion:**

PAR is a potentially valuable tool for healthcare evaluations with the ability to generate nuanced reflexive perspectives and considerations that go beyond identifying the outcomes and gaps in interventions. It draws participants into a reflexive process to define pathways for change. Health professionals identified social inequities as the most significant barrier to promoting early childhood development. These inequities were not addressed in the design and implementation of the early year programme under study. The study supports the need for a multi-level, multi-systems and intersectional framework for place-based public health programmes to have the desired impact and reduce inequalities in access to early years interventions.

## Background

Ensuring a healthy start is critical for good early childhood development [1]. Recognising this as a key priority for public health, the World Health Organization (WHO) published first guidance on the topic in 2020 [2]. While highlighting multiple areas of care and development, the WHO distil their recommendations into four key domains of effort to best support child health: responsive caregiving, promoting early learning, integrating caregiving and nutrition interventions, and supporting maternal mental health. In parallel, recognition of the role of early childhood in adult and population health has spurred the production of a wealth of scholarly conceptualisations of health promotion strategies [3–5] and the development of early years interventions (EYIs). EYIs typically aim to target one or more of the priority domains identified above, with high variance in the strategies and initiatives adopted in countries. For example, in the United Kingdom (UK), child nutrition is promoted through clinical guidance (such as the National Institute of Health and Care Excellence’s guidance on ‘Maternal and child nutrition’ [6]), charitable campaigns like the ‘Baby Friendly Initiative’ [7], financial schemes (e.g. the ‘Healthy Start’ voucher scheme supporting parents with young children to purchase healthy food [8, 9]) and a deluge of educational material for parents and carers.

Other EYIs apply a more expansive and holistic model addressing multiple domains of development, for example, England’s ‘Sure Start’ Children Centres which included child health services, pre- and post-natal classes, additional parental courses, childcare and education [10]. A more recent initiative in the UK is the ‘A Better Start’ (ABS) programmes. ABS promotes good early childhood development through place-based partnerships fostering service development in specific regions of the England [11]. The clinical and development foci of ABS is premised on the ‘first thousand days’ concept, which denotes the period from conception to around a child’s second birthday – “a crucial window of opportunity to improve child (and population) health” [12], and thus centres on family-based health and social care services from the early post-natal periods and throughout the child’s early years, messages subsequently echoed in ‘The Best Start for Life’ report published by the UK government in 2021 [13].

Ascertaining effectiveness of early years interventions and similar public health initiatives that involve multiple projects tackling different (clinical as well as social) domains of childhood development remains challenging. There is a paucity in methodological detail in the literature, and approaches are particularly scarce for evaluating social interventions [14–17]. Furthermore, impact remains difficult to establish due to such initiatives being beyond experimental ‘control’ and particularly in the case of place-based programmes or centres, where assessments likely need to be ‘tailored’ to the local objectives and structures. Evidence of what works is largely either descriptive or focuses on interventions for specific domains of development [18]. A deeper understanding of valuable evaluation approaches for these programmes is warranted.

In line with the complexities of early childhood public health programmes, evaluation approaches which are multi-faceted, taking a ‘system-wide perspective’, which goes beyond evaluating the intervention and explores the systems and context in which programmes are delivered [15] may be helpful. In a review of evaluations of public health interventions, McGill and colleagues identified that while evaluations incorporated a range of methodological approaches to reach their intended aims, few of those were focused on evaluation of *impact* further resonating with the challenges discussed above. The review also revealed that many studies fail to capture the dynamic changes and complex systems perspective, describing static systems at a single time point. Place-based EYIs that are located within a specified area and intended for a selected community, responding to local needs with a focus on partnership working [19], such as ABS, may arguably be even harder to evaluate. These initiatives often have a vast pool of potential beneficiaries and are diverse with regards to their aims. These aims are constantly evolving as local needs emerge and new interventions are added, with new outcome measures and performance indicators. Multiple methodological stances, and in particular adopting an ecosocial approach [20] that can support this analysis, may therefore benefit the evaluation process [16]. A realist approach with more adaptive research design may be required to examine specific interventions, contexts and populations in a system undergoing rapid changes [21].

Urging for qualitative approaches for such evaluations, Deas and colleagues call for the centring of *key actors* in evaluation processes. Authors demonstrate the use of semi-structured interviews with academics and senior NHS staff in evaluating a public oral-health intervention in Scotland [22]. The authors critically reflected on the value of their data, which could provide “a description of the often-hidden processes through which such an initiative can be developed within a described context” [22, p.7]. Privileging the voices of the key social actors and stakeholders is valuable for understanding the nuances and complexities of such programmes.

Participatory action research (PAR) enables “collective, self-reflective inquiry that researchers and participants undertake, so they can understand and improve upon the practices in which they participate” [18, p.854] and can facilitate exploration and analysis of local knowledge [23]. Utilising PAR as an approach for evaluation therefore presents an opportunity for a transformative and nuanced analysis of the impact of public health programmes, particularly when they are place-based and community-centred. Despite this potential, there are few documented instances of its use in evaluating the impact of interventions.

The research reported here outlines use of PAR with service providers to examine the perceived barriers and challenges to facilitating good early years development within the ‘A Better Start Southend’ (‘ABSS’) initiative. ABSS is a place-based public health programme in the southeast of England focused on improving access to and quality of early childhood health. ABSS is one stream of the ‘A Better Start’ (ABS) national programme and provides free services to families with babies and very young children (age 0–4) in the six most economically deprived neighbourhoods in Southend-on-Sea. ABSS has offered major system change across Southend, with thirty one free projects, programmes and activities for local families which are targeted at giving children “the best start they can have” [24]. ABSS places strategic focus on working with local parents and carers to develop and design services. Its projects can be described as supporting access to services and development in the following domains: diet and nutrition, social and emotional development and language and communication. Through this, ABSS ultimately aims to improve the longer-term life chances of its children.

The purpose of the study therefore was to evaluate the ABSS programme, utilising innovative PAR methodology, to specifically explore the perspectives of health and social workers delivering the programme on the barriers and challenges to delivering equitable, good quality services to the community.

The aim of this paper is to present a critical examination of the operationalisation of PAR for evaluation, using some key findings to provide context.

### Regional context

The region of Southend is a highly socio-economically deprived coastal community in England, with twenty-four of its one hundred and seven lower super-output areas (which comprise local neighbourhood boundaries) among the 20% most income-deprived in England. It has an internal disparity of 45.5% points, meaning across the region, neighbourhoods are highly heterogeneous: some are considerably wealthy whilst others are considerably poor. It has an increasingly diverse population, with 12.5% of residents identifying as ethnic minority, and 7% having a national identity not associated with the UK [25]. The region is similarly comprised on seventeen larger political ‘wards’; citizens living in the six most deprived wards of Southend can access ABSS services. Resources were strategically allocated to foster good child and population health in areas at most risk of poorer outcomes. It is in this context of known inequitable access to health that the ABSS programme was implemented and thus evaluated.

## Method

This research was undertaken as a discrete component within a larger summative evaluation of the ABSS programme undertaken jointly by an independent contractor and the University of Essex. The evaluation aimed to determine the impact of the programme of child and family health and wellbeing and community resilience. The PAR component was led with staff – health and social professionals- from different ABSS projects to document changes, learnings and key barriers in meeting the programme objectives and reaching disadvantaged groups.

Participatory evaluation is a powerful approach to enhance organisational learning that can result in better informed decisions [26]. It is an applied social research approach that involves the stakeholders of a programme, including those with programme responsibility, in the evaluation process. For this component, PAR was utilised to examine the challenges and facilitators to promote early years health in Southend, and evaluate the ABSS programme for its effectiveness in supporting this goal, equitably?

We utilised a range of methods including spidergrams, participatory mapping of idealised care pathways and photovoice to elicit perceptions on the barriers and challenges to delivering ABSS programmes and achieving the desired outcomes. These participatory methods offered a unique avenue to understand the programme of work led by ABSS. This qualitative methodological approach was selected as it helps better understand lived experiences from an emic or insider perspective and can be used to “maximise participant’s control over the production of knowledge” [18, p. 4]. Indeed, PAR is especially valuable in exploring areas of health where there are varied and intersecting factors related to disadvantage [23], presenting a very valid approach for tackling the research at hand.

### Participants

At the outset, PAR involved virtual meetings and focus groups with ABSS service managers and programme leads. These aimed to support the researchers to know more about the work of ABSS, build relationships, clarify the project’s ambition, and establish working practices. These were also leveraged to support recruitment of ABSS programme delivery staff to the subsequent phase, who were identified as key actors in the initiative, and experts with valuable knowledge of both the services as well as those utilising these services. Ten programme delivery staff members, representing the typical workforce within ABSS, holding various professional and clinical roles across ABSS programmes were recruited. Our sample included speech and language therapists and assistants (*n* = 4), health visitors and assistants (*n* = 3), support workers (*n* = 2) and nurses (*n* = 1). All participants were living and working in the community and supporting community members in diverse ways. The focus of the PAR activity with programme staff and their experiences complemented other strands of the wider evaluation which focused on perspectives of service users/ community members through surveys and interviews. The findings from those is beyond the scope of this article. Engaging staff members offered a longer-term and systems-perspective on the programme as staff could bring together their collective experiences working for extended periods with multiple and diverse families in the community who were receiving support from ABSS.

Participants were invited to attend four two-hour workshops over a period of 12 months. Overall, twelve workshops were held, four workshops dedicated to each of three workstreams which related to the three developmental targets of ABSS: communication and language development (CLD), social and emotional development (SED) and diet and nutrition (DAN). Participants were not involved in the study design as this was an evaluation. However, once the PAR groups were formed, they were asked to opt for the method they wished to engage with over the evaluation period and given the option of journalling and photovoice.

The first workshop was critical to orient participants to the study and build trust between researchers and participants. It was achieved by clarifying objectives of the evaluation, understanding their current roles, discussing the importance of anonymity, confidentiality and a safe space to share their vision for their respective programmes, and collectively identify bottlenecks and solutions. Workshops took place in a neutral, community centre where ABSS activities were often held, signifying a safe space for participants. Researchers sought to fully understand the participants’ individual and professional context, and reciprocated with transparency around the roles of the researchers and aims of the evaluation. Trust was further enhanced by the pro-longed engagement in the project, where researchers and participants met and exchanged emails for up to 12 months. Two to three researchers were involved in the workshops. At the time of data collection, all were employed in an academic institution with substantial years of research and evaluation experience and led by a professorial level researcher. Prior to the data collection, researchers were not known to participants though familiar with the ABSS programme more broadly. Reflexivity among the researchers, as well as the participants, was facilitated through frequent and ongoing discussions around the points raised in conversation especially where more challenging and critical issues were highlighted and probes about perceptions and experiences of the methods used.

In addition to the PAR workshops, the research team attended two ABSS community events where the PAR outputs were displayed, offering community engagement opportunities. During these, we sought to interact informally with parents living in Southend as well as ABSS ‘parent champions’ to reflect on the findings collaboratively. Parent champions are self-selected ABSS beneficiaries who have taken on more active roles within ABSS, such as joining programme committees or steering groups, or initiating new sub-projects.

### Data collection

Workshops were held in community settings (for example, in the local library) with PAR participants to support data collection. The initial workshop focused on establishing working practices and establishing a common understanding of the issue [27]. Alongside traditional qualitative methods such as focus groups, creative approaches were utilised to empower participants to exchange authentic knowledge. In doing so, the providers were situated as ethnographers [28]. Throughout all activities, researchers made field notes to complement transcripts and other outputs.

Initial knowledge about ABSS, the services offered and barriers families’ experienced to accessing them were developed through spider grams [29] and mapping of care pathways. Spider grams are visual tools often used to collectively brainstorm and record discussions, whereby important components, issues or questions can be identified, and answers or solutions deliberated upon. They are used flexibly in qualitative research, and considered especially useful for when issues can be broken down into smaller parts [29]. We asked participants to draw their specific programme/service, its core components, and record the main barriers in delivering their roles and meeting the objectives of the ABSS programme. They were asked to consider the programmes they deliver and identify the barriers, and the subsequent discussion of each was captured as branches on the spider gram, which comprised more detail on the issue, different facets of the issue and possible solutions. This more generic discussion on their roles and key challenges was expanded and given more focus by introducing the service mapping activity [30] – a form of asset-mapping, commonly used in community-based PAR work [31]. The activity, detailed below, enabled greater reflection on the rationale for the service, the underlying assumptions and resources necessary to deliver its different components.

Participatory mapping is used in varied and creative forms across fields, but broadly describes an interactive visual method to tackle specific questions, which facilitate a process of description, to elaboration, to theorisation which can complement traditional verbal approaches [30]. For this study, a modified technique was used whereby participants were tasked with visualising their associated service pathway and to reflect on what they consider their ideal service pathway for achieving the objective of their respective workstream would be, if resources were not a constraint. This visual activity aimed to understand what practitioners wanted to see from their service and why, and empower them to hypothetically design the care pathways that would achieve success and good healthy child development. The pathways also stimulated reflection on the policy, system and other challenges and barriers that did not allow services to be delivered in this ideal manner.

Following the first workshop which helped generate a reflective account and critical overview of the programmes, participants subsequently engaged in photovoice. Photovoice is an established participatory action research method [32] used to help gather information about people’s views of their own lives [33]. It involves participants taking photographs of objects, spaces or places that are meaningful to them. In this study, photovoice was used from participants’ positions as practitioners working closely with families, both as a close observer of people’s lives and as a direct witness to action taken in those communities to improve lives. Photovoice was particularly appealing to participants and deemed relevant for assessing the ABSS programme as it is rooted in the ideas of promoting social justice and influencing policy change [34].

In this study, participants were asked to take photographs of ‘resources’ (objects, services, spaces or places) that can be perceived as enabling or hindering the health of children and families living in the ABSS wards, vis-à-vis the programme objectives. An amended version of the SHOWeD framework structured these sessions [23, 33].

In addition, the researchers collated field notes from discussions with eighteen parents, five parent champions and seven community service providers engaged through the community festivals/events organised in the dissemination phase.

### Data analysis

Data took various forms – textual (audio transcripts of recorded conversations/ focus group discussions, field notes) and images (spider grams, service maps, photographs). As such, analyses were complex. Tools and outputs utilised across the workshops (e.g. spider grams, service maps and groups discussions on photovoice images) were synthesised by participants and triangulated by the researchers. Photovoice inherently involves a dialogic approach to analysis with and by participants as well as researchers. The final photovoice workshop underlies this, where participants identify the most pertinent images and conduct a collective analysis, identifying themes and curating the photovoice exhibit which involves an accompanying narrative, and selected illustrative quotations. Additionally, member checking was involved throughout. After each workshop, participants were emailed the summaries and themes discussed by participants and asked to feedback on their suitability and recommend changes to the analysis. Participants were directly involved in the analysis and design of ‘actions’ including curating a ‘photovoice exhibit’, which could be displayed in community events and used to engage community members. In assembling the photovoice exhibit, participants selected photographs for inclusion, the themes they addressed and the narrative that best described them. The exhibit, a key output of this study, was used in community events to initiative dialogue, forming the main form of dissemination. The themes and priorities perceived by the ABSS staff were further validated and corroborated in conversations with parents and carers (community members) through these community events. Informal field notes from community events aided in identifying priority challenges experienced in the region, though we recognise that a limitation of the method as implemented was the lack of involvement of family/carer service users as participants in the PAR activity itself. Further extensive analysis was undertaken by the researchers, drawing on the analytic framework for photovoice outlined by Tsang which indicates a process of researcher analysis, participant analysis followed by cross-checking and finally theorisation [35].

All workshops were audio-recorded and transcribed and were used to triangulate data from focus group discussions and the photovoice theory, for a more detailed analysis.

For this, NVivo software was used. Transcripts from all workshops were disassembled and coded, then reassembled into themes which then comprised a set of codes, drawing on thematic analysis approach [36]. Some codes were inductively influenced by photovoice themes developed by participants in the photovoice, though within the confines of the scope of evaluation (that was aimed at examining implementation barriers). 

It is pertinent to note that participants were not directly involved in the writing of this manuscript, though they were consulted and invited to comment on drafts on an executive report that contained the key findings. Attempts to seek participants’ feedback on the manuscript were not successful. Many email addresses were inactive, indicating the high turnover of project staff, or the finite nature of their role on time-bound projects.

### Ethics

Ethics approval was granted by the University of Essex’s Research Ethics Committee 2 (Reference: ETH2021-1297). The research governance contact in each NHS Trust that employed potential participants was asked to give approval to include their staff within the participatory action research. The study was conducted according to the guidelines and requirements within the University’s Code of Good Research Practice and this article conforms to the “Standards for Reporting Qualitative Research (SRQR): 21-items checklist” [37]. Participants were sent information about the study before the first meeting, and then were provided with a participant information sheet at the start of the first meeting. Informed consent was gained in writing before the start of the discussion.

## Results

In this section, we first provide a summary of the key themes from the evaluation. These are further discussed throughout the second section, which focuses on illustrating the operationalisation of PAR.

### Summary of key themes

The application of PAR yielded critical providers’ perspectives on key challenges of delivering these programmes, and the factors that impeded their uptake in the community, and hence effectiveness. Challenges often related to social determinants (including socio-economic circumstances and the cost-of-living crises, housing, English language status) and commercial determinants (including unhelpful marketing tactics from the ‘baby food’ industry). Parents and carers corroborated these findings in the community events, which are also incorporated in the discussion below.

### Operationalising PAR

To illustrate our application of PAR in this evaluation, we draw on Cornish et al.’s (2023) four principles of PAR: Experience, Knowledge in Action, Collaboration through Critical Dialogue and Transformation [27]. We explore each of these in turn to describe the process of engagement, how these were encountered – individually and collectively - in our participatory evaluation project, and what knowledge such engagement generated.

#### Principle 1: experience

‘Experience’ here characterises the ‘authority’ of experience and observes the value of expertise gained through it [27]. In this evaluation, participants reflected upon and articulated accounts of their interactions and experiences with local families about their babies’ development and the challenges with accessing services.

**Fig. 1:**
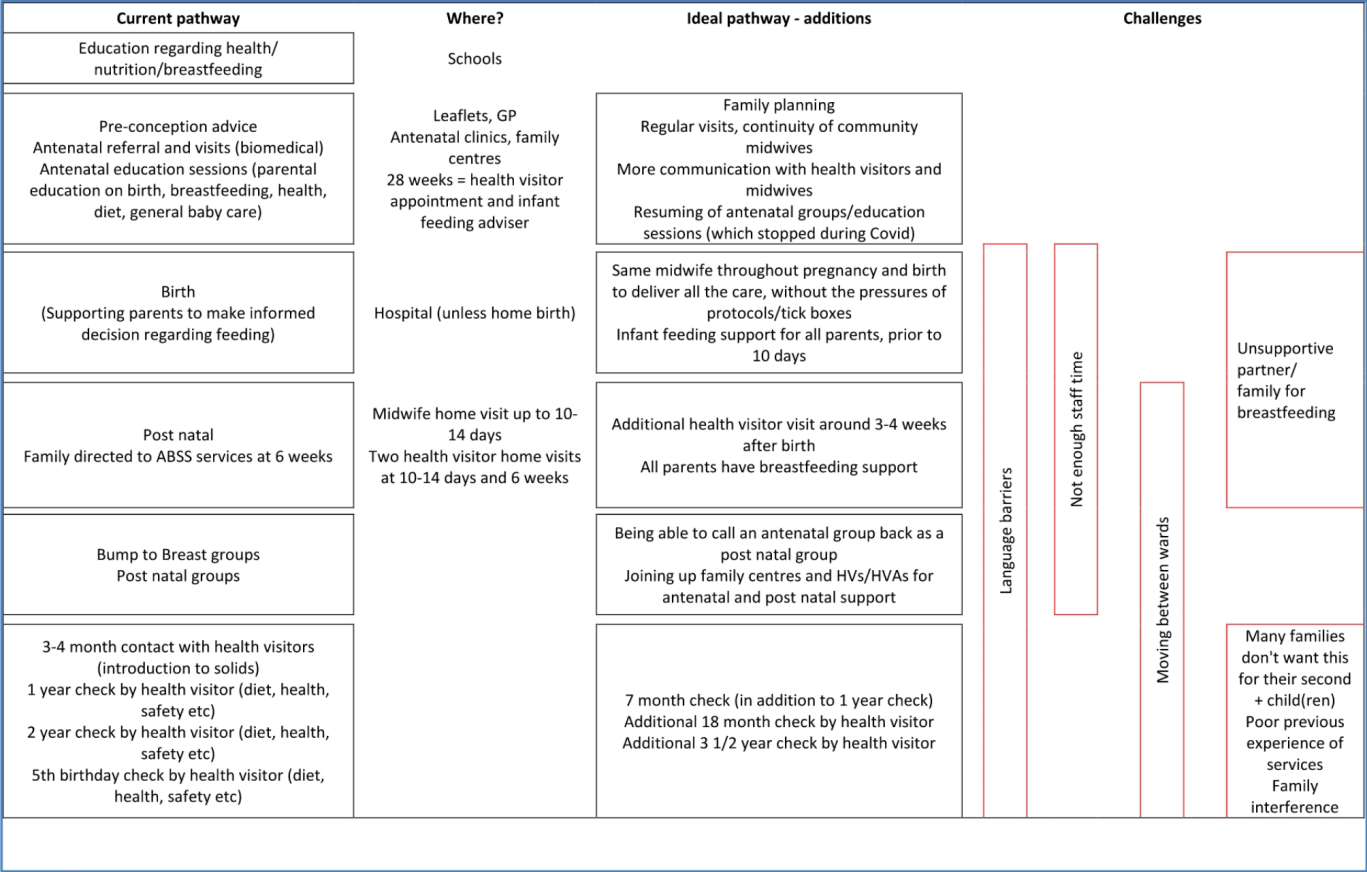
Output from DAN workstream service mapping activity

The service mapping activity elicited valuable knowledge about current practices and participants’ perception of existing pathways of care. Drawing on their expertise, participants were asked to draw ‘ideal’ pathway of care and reflect on how current practices departed from what they considered *should* be in place to maximise equitable access to their services. This reflexive process enabled participants to reflect on their experiences of delivering care in the community and identify the problems / barriers experienced by the community and discuss potential solutions to some of these issues. For example, as illustrated in Fig. 1, in the ideal care pathway for diet and nutrition participants recognised that the parents were not making fully informed choices about feeding for their child and identified that additional health visitor checks were needed to address the gap. This was accompanied with acknowledging ‘not enough staff time’ and ‘human resources’ as a key gap to ensuring parents and carers are receiving appropriate advice and support for nutrition. Therefore, the activity invariably moved between exposing limitations in their autonomy in practice (restricted staff, limited health visitor checks) and empowering their autonomy through proposing solutions.

The figure contrasts the current service pathway (on the left) with the ‘ideal’ pathway practitioners want to see for their respective services (on the right). It accompanies a description of necessary interventions to close the gap, and where and when such intervention may be required. On the far right, participants outlined the challenges with each component of the pathway.

While reflecting on their experiences in discussion about barriers to provision and access, participants identified a range of social, political, economic, and commercial determinants as limiting access to EYIs and inhibiting good childhood development. Participants across all workstreams, but especially in the DAN workstream, highlighted risks associated with food poverty and the growing cost of living crisis, highlighted in the excerpts below from two participants in this group:

*“The main issue that we are finding is food poverty. People just can’t afford to buy formula. You are probably going to go through a couple of tubs a week, which is at least £20.”*

*“I’ve seen a mum recently who physically hasn’t been able to breastfeed. But financially she can’t afford to buy formula either. Where do you send them if the food banks aren’t going to have the formula there?”*

When elaborating on these concerns about the cost of living and rising food prices, participants described practices that reflected the intimate nature of their role in supporting the families, which extended beyond health advice and support to discussions around finances and parenting decisions, sharing low-cost recipes – placing them as confidants and valuable knowledge brokers in this context.

*“When we are talking to people*,* if I’m talking to someone about formula*,* I will ask them*,* “Do you know what formula costs? Are you able to afford it?” Because at the moment with the costs of electricity and gas*,* some people actually can’t. So*,* these [are the] questions that we’re having to ask.”* DAN workstream participant.

Here, participants reflected particularly on ‘low-income’ families’ and their interaction with commercial ‘baby food products’. One participant argued: *“It can’t be the parents with no money [buying the products]*,* they can’t afford them.”* which was countered by another participant stating: “*Do you know what*,* I actually see the opposite*” before going on to explain:

*“They [the parents from low-income households] will sacrifice something else to buy the products*,* God forbid… but get it*,* in any other way that they can”* DAN workstream participant.

A practitioner added:

*“Well*,* they will give normal cow’s milk. They’ll start solids early. They will water down milk.”* DAN workstream participant.

The commercial influences thus appear to be mediated by the socio-economic factors, compounding attempts of EYIs to support childhood (and family) health and wellbeing. This was echoed in our informal conversations with a parent at a community event who reflected on the ‘hidden’ economic costs of bottle-feeding including bottles and sterilisation equipment, and how parents may often not have the full information on the health effects of bottle feed and other supplementary foods in the market. The participatory nature of the data collection here enabled intimate knowledge of limitations in the ABSS services, the complexity of ABSS beneficiaries’ lives, and their perceived challenges to be shared, debated, and understood by both participants and researchers.

#### Principle 2: knowledge in action

Participants brought together their diverse experiences to the PAR workshops, and through an exchange of these learnings in a process that combined sharing, evaluating and applying these to practical contexts, generated new knowledge. Here, knowledge in action was observed and reflected upon at both the individual and group levels. One example of this process is in the quote below. The participant, in identifying potential barriers to accessing learning and development services, simultaneously reflected on how they mitigate these barriers, while also acknowledging the limitations of their mitigation strategies. The excerpt below illustrates this process.

*“Sometimes parents may have other things going on in the background*,* whether it be housing*,* their own mental health*,* or domestic issues*,* which are taking more of a priority than their child’s speech and language*,* understandably. That would be a big hindrance for a family accessing our services…Again*,* we do try and support that if we are aware of things*,* by offering a home visit… But again*,* sometimes the issue preventing a family from accessing is so great that even that isn’t going to work at that particular time in the child’s life.”* CLD workstream participant.

In this example, we observe the practitioner firstly sharing their knowing about vulnerabilities of specific populations who may not be able to physically come to seek services. They also offer reflection on how home visits could counter this; but draw on their experiences of home visits to ultimately trigger a discussion on broader social determinants that may render home visits ineffective. Citing their experiences of visiting homes of some families/ mothers/carers, participants shared how those most in need of their services are often living in damp, mouldy and cramped social housing. These conditions not only affect social development of the child but also limits the potential benefits home visits can have. This analysis emphasised how social environments may exclude already marginalised communities from public health initiatives such as ABSS, therefore contributing to a widening inequality gap. Echoing this finding, a conversation with a local parent at a community event highlighted specific barriers for them as someone living with a physical disability (and a wheelchair user) raising a child. They shared that parent-child groups which, even though held in community spaces, were not always step-free or wheelchair accessible. Thus they, and people in similar situations, “feel like they cannot be bothered to leave the house because it was too much hassle, or dangerous”. They, however, acknowledged it was important for their child’s health and wellbeing to be going outside and engaging with the community, thus signalling a complex and nuanced compromise that parents facing additional barriers experience in giving their child a healthy start.

**Fig. 2:**
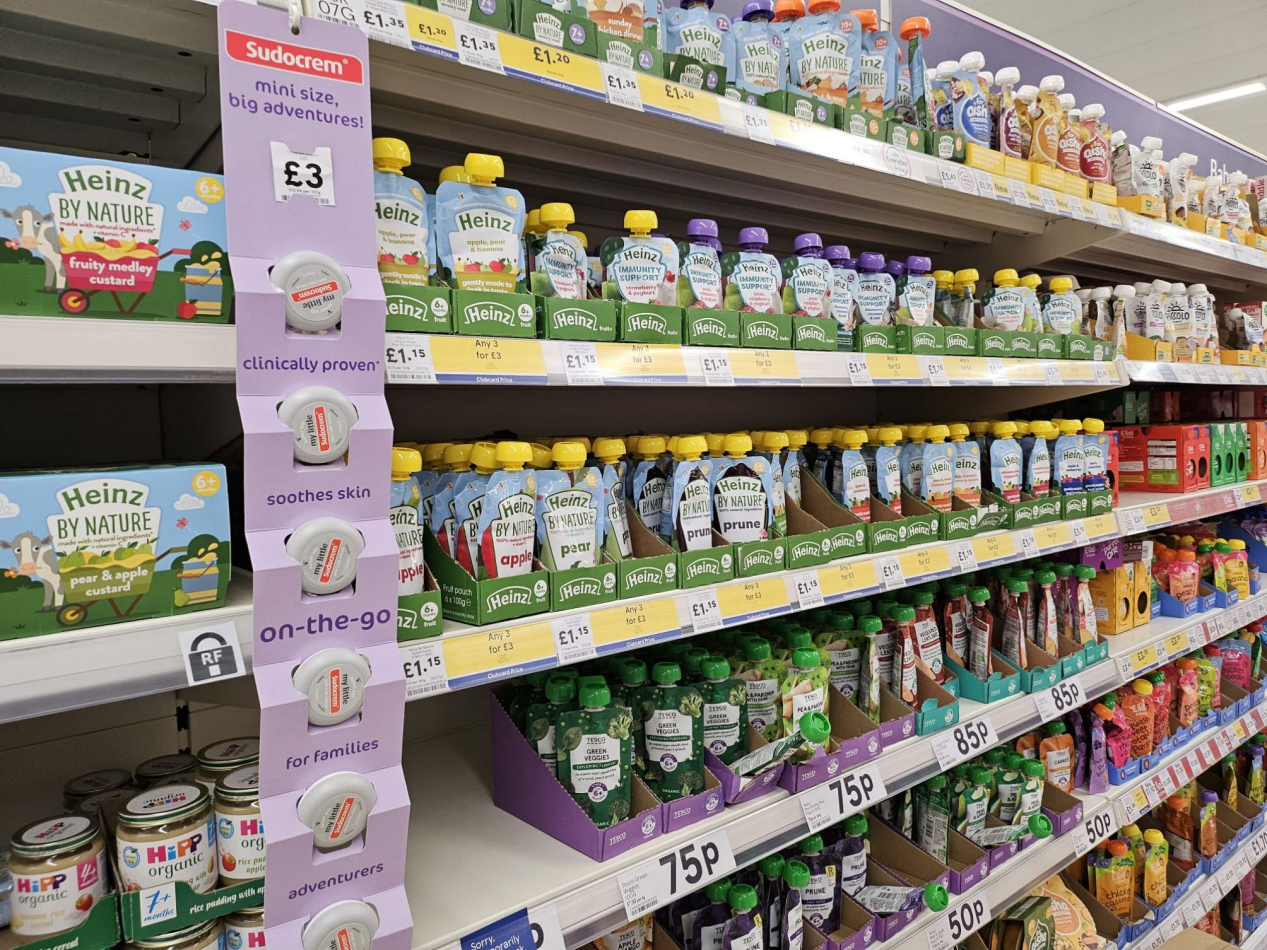
A representative photovoice image from the DAN workstream. (Disclaimer: the original image was too low resolution for publishing, the authors have sourced a close alternative)

Knowledge in action was evidenced throughout the PAR process, as participants applied their knowledge to identify solutions and actions that can be taken to improve the services they offered. The below excerpt of a conversation was stimulated by an image of ‘baby food’ products and a supermarket aisle, produced by a participant (Fig. 2).

The image depicts supermarket shelves stocked with a wide variety of ‘baby food’ products, highlighting the popular ‘pouches’ which were identified collectively by the group as particularly common, but problematic, foods.

The image was taken to illustrate the abundance of commercial baby foods in market aisles. It triggered a dialogue among group members, on analysing the role of supermarkets in normalising baby-food market consumerism. Having identified, in earlier discussions, the health-risks of baby food products and their unaffordability, two participants in the DAN workstream reflected:

*P1 “That photo was literally just, again, to show the overwhelming amount of baby products that are on the shelf. And I mean, that was just one side. The aisle was both sides, with nappies and things like that as well. But one side was all food and formula, and it just strikes me straight away that if you are a parent and especially a first-time parent, and you walk in and you see all of that, straight away you’re going to go, “Oh, that’s normal. I could use that”*.”

*P2 “They make sure you’ve got to go down the aisle, when actually you could take all that away -even the formula - and not have to rely on that to feed your baby. If you think about it, if that was removed from a supermarket, you could still feed your baby from birth without all of that.”*

*P3 “But have you noticed as well that in the majority of shops, the baby food products are in the same aisle as things like the essentials? Like nappies and baby wipes and that kind of thing. So then literally from birth or before birth, a lot of parents buy those ‘essential’ products* (commercial supplements) *and then only later see other natural foods.”*

One participant then reflected:

*“If the nappies and essentials were in a different aisle (than that of essential products), parents wouldn’t be under pressure from such an early stage because they could choose not to go down that aisle.”*

In this instance, the discussion helps to identify commercial sector and supermarket giants as key actors, their harmful marketing practices, and potential areas for action (placement of products) in curbing some of the harmful influences of the industry. The alternatives identified could create greater space for parents to make choices for their child’s development. However, in discussion with a parent at a community event, the importance of peer-influence was also raised as they commented that they considered other parents to be the largest influence in deciding what to feed a child, but those parents who are “less-informed” are the ones more likely to be vulnerable to buying what is commonly advertised (such as the convenient snack foods). Besides illustrating the influence of commercial practices, this example reveals how PAR can be a conduit for participants to elucidate complex factors at play. These factors limit the effectiveness of EYIs by normalising the purchasing and consumption of non-essential, unhealthy and expensive commercial foods that stand in conflict with advice from public health professionals for promoting healthy growth and development of babies.

#### Principle 3: collaboration through critical dialogue

This principle emphasises the importance of dialogue in “harnessing the diverse sets of expertise and capacities” [27] and establishing meaningful collaborations between its participants. By leveraging the participants’ emic perspective and expertise and centring on collective discussion and reflection, PAR enabled the production of a nuanced account of the multiple challenges impeding effectiveness of the intervention.

Emancipatory dialogue across and between service delivery staff who were unlikely to have otherwise met, was powerful in the exchanging, creating, validating and critically evaluating local experiential knowledge.

This process produced rich and insightful data, which participants made sense collectively, as has been described above – reflective of a transformative praxis of collective critical consciousness” (33, p287) raising [38, 39]. For example, participants in the CLD workstream discussed what engaging in photovoice enabled:

*P1 “Had we not had the pictures, I don’t think we’d have necessarily come up with as much stuff that we have or have had the discussions we have had.”*

*P2 “Yeah. It’s allowed some really good, valid, creative discussion points from different perspectives as well.”*

*P1 “I’ve learned a lot about you guys.”* [referring to other participants and their work streams]

This research process enabled access into the relational pathways through which the emancipatory aspect of PAR can come to the fore. PAR allows for new possibilities through the recognition and engagement of others in dialogue, drawing on individual and collective experiences to generate meaning [23]. In this process, participants agency through the relational work they engage in, becomes a means of ‘making and re-making’ sense of the context in which they deliver services [28]. For example, they acknowledged the wider structural determinants of health and raised concerns about the disconnect between this knowledge and their professional role, despite its impact on populations’ access to the services they deliver. This highlights the need for a praxis that engages health workers in navigating the impact of public health programmes, being in proximity to, but also protected from, precarity and social harm (as illustrated through earlier excerpts) [40].

Engaging in this critical reflexive enquiry organically directed participating practitioners to emphasise the social and commercial environments as barriers to child health and well-being (beyond a narrow focus on the early years services they provide). Collaborative dialogue also signalled the need for an intersectional approach to be considered in future place-based EYIs. The latter was supported by the recognition of multiple levels and layers of disadvantage that were frequently discussed in relation to accessing health and health services. Participants often described the challenges faced by those in the community who represent multiple and compounding structures of disadvantage, for example young single mothers who may have grown up in the care system, who are also dealing with trauma and mental health difficulties, transient populations such as refugees and asylum seekers, those who are placed in poor quality social housing are violence survivors, or have a child with special educational needs. The value of PAR is highlighted especially in these rich and considered discussions, as these case examples were elucidated – and built upon - directly from shared stories of experiences of our participants. This resonates with Kapilashrami and Marsden’s discussion on the value of using PAR for critical reflexivity and consciousness raising on structural barriers to accessing health enabling resources [23] and the relevance of considering intersectional social locations that disproportionately impact those leading “chaotic lives” (p. 13) and their experiences of injustices.

#### Principle 4: transformation through PAR process

Cornish and colleagues emphasise the importance of PAR in creating “empowering relationships and environments” as central to its methodology [27]. These relationships are enabled through the process of consciousness-raising and collective enquiry of problems and solutions. Opportunities for such transformative processes were evident in our study, demonstrated in the excerpts and analysis below.

A resounding realisation across participant groups when considering challenges in their services was that the programmes they worked on were likely meeting the needs of a narrow population group, unable to reach and further marginalised multiply disadvantaged populations - minority groups and those at the deep end. Through PAR, they collectively reflected upon failures to effectively build partnerships and accessible services for all. For example, in both the CLD and DAN workstreams, participants spoke of how parents who did not speak English as their first language (or have moved to England from a country where public health services are not common) may not even know about services available to them. One participant outlined:

*“And I think the other key issue is*,* how do you know what’s available to you? If English isn’t your first language*,* or if the community that you are being housed with all similarly have English as an additional language. If you’re not a fluent English speaker*,* how can you pick up the fact that you have a health visiting service even?* O*r some of our refugees from Afghanistan*,* for example*,* were saying that they would never go to a health provider unless there was an emergency*,* or their child was ill. They don’t have that preventative service (*in Afghanistan), *so weren’t aware that there was the health visiting team*,* or that any of the concerns that they might have regarding their little one’s development could be supported.”* CDL workstream participant.

Participants across workstreams also critically reflected on the key initiatives employed (engagement sessions, interpreter services, translated materials) to address these access barriers and highlighted their limitations, and/or lack of impact:

*“They (health visitors) invite them (parents) down to a room within the hostel to explain the services. We’ve had quite a few discussions with the health visitor about this*,* but she said they just don’t come down.”* CLD workstream participant.

*“Sometimes the interpreters don’t turn up*,* or it’s only via a telephone link. It’s very hard to interpret still*,* because you can’t see any facial expressions. And just being able to book somebody can be quite difficult at times as well.”* DAN workstream participant.

*“You can translate the communication skills screening tool into certain European languages*,* but other languages not necessarily. There are no words for some of the words we are using. They don’t exist.”* CLD workstream participant.

**Fig. 3:**
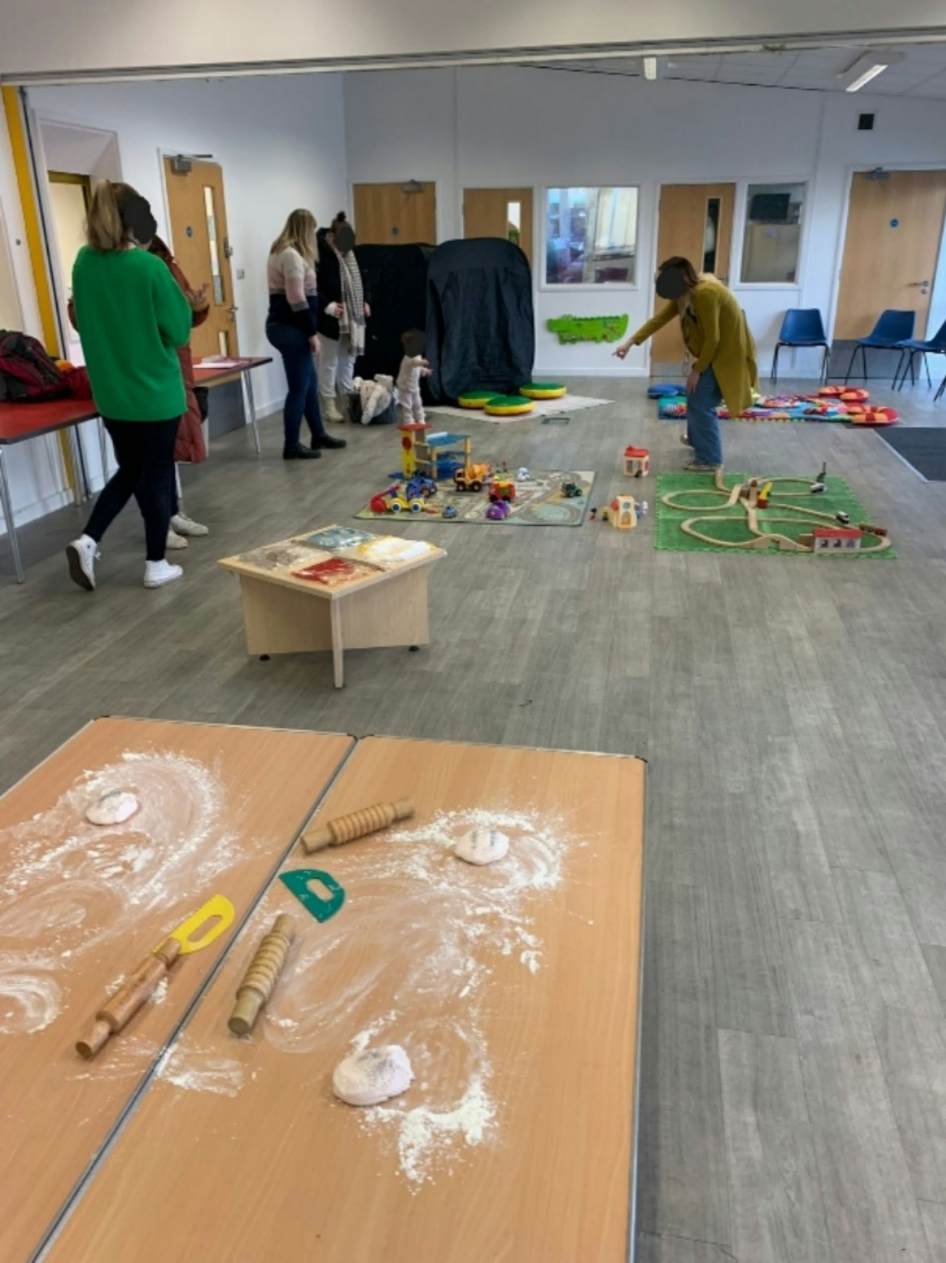
A photovoice image from the SED workstream

We observed participants as a group moved incrementally from ‘challenge’ to identifying ‘solutions’. A notable finding from this was the need for better working relationships between practitioners across different services that were hitherto operating in silos. Being in the collective space that PAR enabled made participants acknowledgment this. For example, discussions between health visitors and midwives led to clear recognition that it would benefit both teams if they worked more closely, and/or had a better communication system between their services. Reflection through photovoice also supported the sharing of innovation and knowledge exchange, as one participant described through their photograph of a session at the Lighthouse Child Development Centre - a local diagnostic centre for children with special education needs (Fig. 3). The image depicted a ‘barrier’ - this was the only service offering diagnostic assessments in the area meaning long wait times, and as such children and families were for a long time not provided with support. The participant subsequently highlighted a successful piece of work they had achieved which provides grounds for a solution to the identified challenge, as articulated in the quote below. This is illustrative of the establishment of empowered relationships, and an empowering environment – central to the process of PAR [27].

The image is of a play session taking place at the of the Lighthouse Child Development Centre in Southend.

*“This is a picture of the Lighthouse Child Development Centre*,* which is the only diagnostic centre in Southend that children with special educational needs and disabilities can go to get diagnosed… It’s a massive challenge for families to get into this centre. The waiting list for appointments are a year or more long*,* so they wait years and years and years to get into this centre.”* SED workstream participant.

*“There’s another thing I wanted to mention about this photo. While taking this*,* we actually went into the Lighthouse as a service*,* and we did a presentation for their staff on what our service is about and how we can assist them and work together. So*,* a positive note to this picture is that we’ve now agreed to work together*,* our service and the Lighthouse…”*. SED workstream participant.

The quotes are also a reminder of the challenges with the ABSS programme in meeting the needs of some of the most vulnerable in the community, and the need for more connected services. The use of photovoice, and PAR approaches more broadly, in stimulating this transformative, positive reflection serves as valuable evidence of the usefulness of this approach in evaluation.

**Fig. 4:**
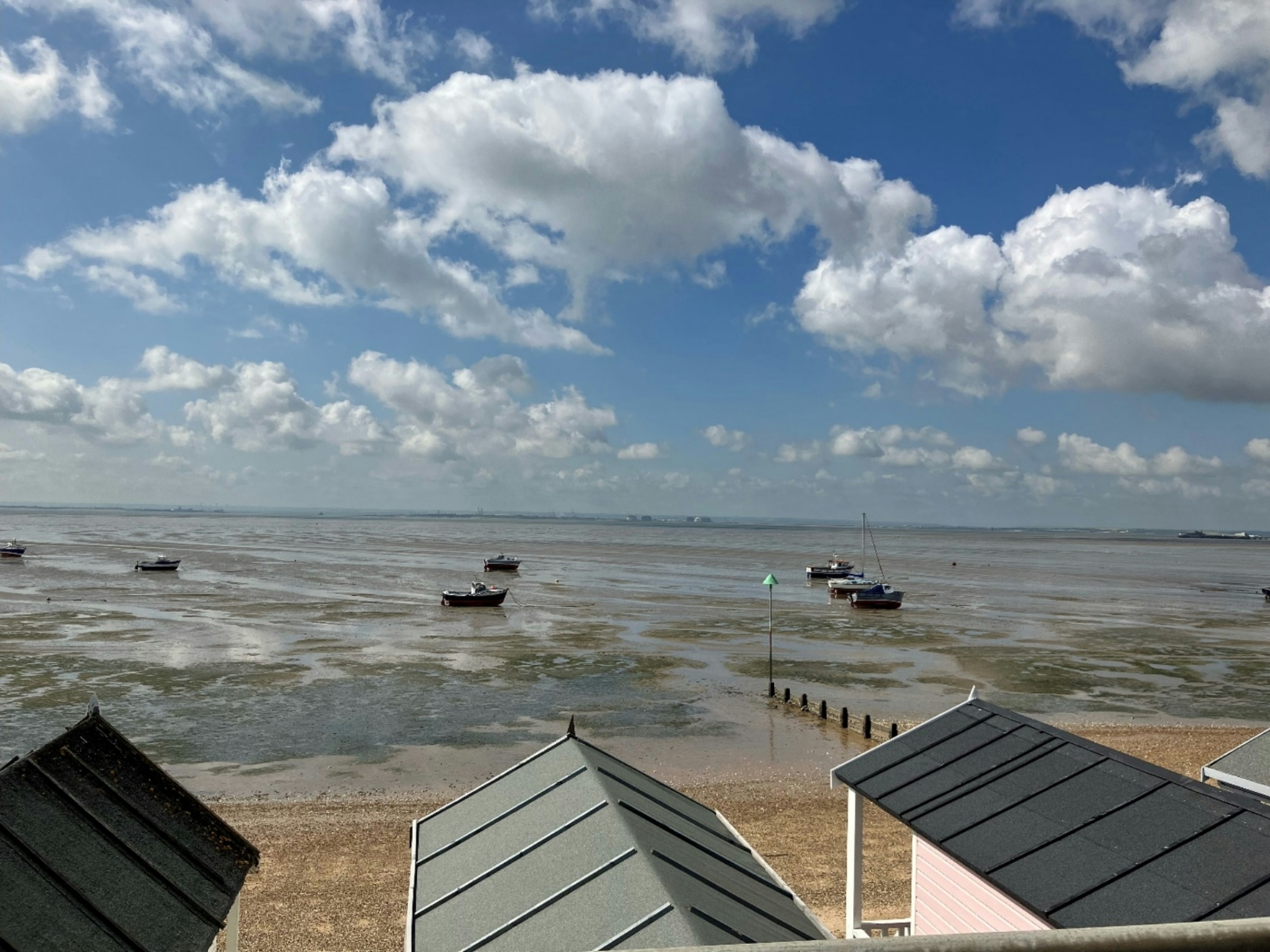
A photovoice image from the SED workstream

The photovoice methodology also offered the space to contemplate nuanced paradoxes in facilitators and barriers to accessing health. With reference to the image provided in Fig. 4 below, a participant in the SED workstream commented: *“I took a picture of the seafront because I feel that Southend is a bit of a double-edged sword.”* They clarified:

*“I feel like in terms of social and emotional development, for some of our families, being on the seafront is a godsend…every Wednesday we do a wellbeing walk and we rotate around different areas… I just thought it was a nice picture to take because I think getting people out and getting them to have that fresh air and just walking and talking is really good for some people.*”

They continued:

*“But I also feel that this is the end of the line for most people. A lot of people get rehoused here…I’ve got quite a few that are on the homeless health visitors… And they’ve been placed in Southend*,* which is away from family support.”*

The photo shows the coastal region of Southend, with a view of the sea. Discussion on the image emphasised the importance of social environment and ‘blue’ and ‘green’ spaces in the development of children and families’ well-being. This was linked to safe and affordable housing and healthy living environment as an important social determinant of early years development but also the role of community and family support. However, despite proximity to coastal areas, and opportunities for community support, participants highlighted the remoteness of Southend and how it is being treated as London’s ‘overspill’ housing create challenges for individuals and their families displaced from their familiar support networks, as well as creating difficulties for them to access public health services for those without fixed addresses. The PAR approach elucidates transformative thinking that goes beyond simply considering the effectiveness of a given programme but extends into a more radical understanding of the interplay between social determinants of health and the intersecting national and local priorities.

One expected outcome of PAR is critical consciousness-raising of systemic issues among participating groups, and the production of “new subjectivities” [41]. Thus, it is pertinent to examine the extent to which participants were affected by engaging in the process. The organic discussions pertaining to social and commercial determinants of children’s health and well-being, as outlined throughout this paper, serve as an indicator that this was at least partly achieved in this instance. PAR participants frequently discussed systemic issues, including housing, economic conditions, neighbourhood deprivation, far beyond their immediate professional concerns. For example, reflecting on the consumption of baby formula, one practitioner from the DAN workstream commented: “*I’m not anti-formula. But what I am ‘anti’ is the amount of aggressive advertising that they push on people*,* and that our government let them”*. In these instances, participants showed a shift away from their identities as health and social care workers, towards social actors reflective of an advocate or lobbyist. Moreover, across the three workstream groups, participants made calls for social change targeted at various agents including the government, the media, as well as commissioners of services. The transformative aspect of the PAR process was reflected upon by participating practitioners. Participants emphasised the depth achieved in utilising PAR methods for analysing challenges. For example, one participant commented:

*“It has pushed you to think about things in more detail. You can bring up so many different and connected topics*,* even when it is a simple picture.”* SED workstream participant.

Reflecting on their experiences of using photovoice, participants’ shared feeling empowered by recognising their professional, implicit knowledge, and enjoying the task of sharing that knowledge and engaging in dialogue with other professionals who they might not otherwise meet:

*“I know that these are the areas that our families are finding challenging*,* a struggle. So*,* it was quite interesting to sort of go out and find this is a problem. It was exciting to share that with others and find their views and experiences resonating with ours”* SED workstream participant.

Engagement in this work offered participants time and space to reflect on aspects of their work and experiences which might not be routine (or even possible) in their day-to-day roles. Upon sharing their thoughts about the method, one participant commented:

*“I think photovoice is a good thing to do*,* because when you’re taking the photos*,* it makes you really think about the photo*,* doesn’t it? Really analyse*,* why is it like that? How can it change? And I think it’s great. It should be done more*,* shouldn’t it?”* DAN workstream participant.

Offering a space for participants to voice and harness their deep and nuanced insights into the ABSS programme, the local context and the experiences of the community, PAR results in transformative experiences and paves the way for critical conversations required for building an action agenda.

In summary, the PAR process and the collaborative analysis that ensued, revealed that while ABSS enhanced access to services and health for many in the community, the programme did not target or mitigate the powerful social and structural factors that impeded uptake. It instead carried a degree of risk that those members of the community who were already most at-risk of poor health and outcomes (i.e. on the margins of society due to their social locations and the structural injustices they have experienced) could become even more disadvantaged thus contributing to the widening of the access to health gap. These findings serve as the beginnings of an action agenda, which focuses on lobbying for greater recognition of these barriers by health and social, education, housing and transport policy decision-makers and public health programme developers.

## Discussion

*“Labelling our work PAR does not make it emancipatory*,* without emancipatory action.”* (Cornish et al., 2023, p. 10) [27].

Cornish presents the fundamental, unavoidable nature of PAR by true definition, and probes us to reflect upon the agency to action evoked in our study. Kapilashrami and Marsden emphasise the importance in PAR of going beyond a critical reflexive inquiry to engage research participants (and local communities) in processes of planning and social action [23]. This often demands amplifying localised action and reflection beyond the groups involved in research and linking them with broader policy and planning networks. In line with this, the findings from this study were taken beyond immediate research participants to managers, health authorities and other ABSS constituencies through multiple dissemination events.

The community events engaged local families in a conversation on the key determinants of early years development, captured in the photovoice exhibit. This not only helped validate our findings but extended an opportunity for the critical voice of parents to surface and partially redress enduring gaps in our analysis of ABSS. Leveraging the perspectives of parents provided space for a “better understanding of the diversity of lived realities” and responded to the imperative for acknowledging and recognising differences within and across community participant groups of *parents* as different to *practitioners*, as well as intersecting access points within these [42]. For example, many of the parents we spoke with at the event were or had previously been ‘parent champions’ of ABSS programmes, and many of our staff participants had children growing up in Southend, which may question how far beyond the dominant social realities our findings really are situated.

A theoretical vantage is useful to seeing and appraising initiatives such as ABSS. As such, PAR offers a pragmatic access point, for theory and practice to meet, and as a result inform praxis [20]. It thus affords an alternative framework to analyse, in context, population health and health inequities as embodied processes, revealing the workings of structural change in our jointly biophysical and social world. The tendency for the PAR process to replicate wider power dynamics, because the group process is not outside of the social reality in which it takes place, needs to be a priority consideration. Therefore, the challenge for taking an intersectional approach to PAR is how to recognise and directly engage these dynamics within the research process [42] as well as a commitment to attend the conditions necessary for meaningful participation in the process of PAR. It is worthwhile to note that discussions throughout the PAR rarely raised any kind of community-level or personal ‘deficits’–in Mothering or parenting styles or abilities–as barriers to accessing good child health, which is contrary to much of the “framing and taming” of EYIs in general [43] and more challenging of the status quo. This again possibly highlights the nature of PAR to enable a context that is cognisant of power differentials, in a multi-layered analysis [44].

Ongoing work to take this action agenda to stakeholders is planned, for example, community centred dissemination events and conversations with programme directors to inform future initiatives. Furthermore, recommendations will be produced for commissioners of place-based programmes, health professionals delivering these, and directors of future programmes with imperatives on how to enhance access to health, taking an intersectional approach – focused on that which avoids reproducing power asymmetries, ensures inclusivity and ultimately strives towards a reduction in health inequities [23]. However, the short-term nature of this evaluation project, and indeed the ABSS initiative (itself limited to 5 years of funding), mean that change may not be observed in the lifetime of the project – when it terminates, staff and services are decommissioned. This presents a limitation of the evaluation, and indeed presents a challenge to using PAR as an evaluation tool, especially when applied to time-restricted projects, even if there are longer term benefits such as developed collective agency and newly acquired skills by individual community participants, and/or the researchers [27]. In this regard, PAR can be considered a powerful tool within a toolkit for evaluation.

There were some limitations to our research and as reflexive inquirers it is essential to document these. Crucially, the PAR methodology applied in this study must be scrutinised with regards to who was ‘in the room’ producing the knowledge, and importantly, who was not, and the implications of this for the findings of an evaluation. In this component of the evaluation only a small number of ABSS programme delivery staff participated, and therefore these findings are centred upon a circumscribed (though, at least across our set of participants, seemingly common) set of experiences and knowledge.

This was partially offset by using community events (e.g. community festivals) to reach local parents, other service providers and community members. Here, the photovoice exhibit co-designed by the practitioners and researchers was used to engage parent champions and community members to corroborate findings and capture any departing and dissenting views. Yet, there is need for defining a greater scope for co-researching and co-producing actions for change, with both families and providers, ensuring greater diversity of experience and capacity for transformation and action. This can substantially aid authenticity to the principles of PAR and intersectionality research.

Further, the extent of the (partial) transformations experienced and shared by our participants, as articulated in the previous section, beyond the ‘participatory space’ is unknown, but it is our hope that these “live and survive” beyond the PAR work [41]. Engaging policy decision-makers – particularly local commissioners in this case of ABSS where, due to the time-limited funding, there is a very real threat of substantial service redesign - in this work may have supported this ‘social mobilisation’, and influenced changes addressing the participants’ agenda and priorities [23].

Another dimension to reflect on is the gendered roles intersecting with our topic of research. This concerns the professionals that participated in the PAR (who were all female health and social care workers, many of whom lived locally, with their children), the clinical targets of the ABSS interventions which concern roles that are predominantly regarded as ‘women’s roles’ (e.g. postnatal and infant health, feeding practices, child-rearing and social and emotional development), as well as the all-female research team. This context is however also representative of the gendered nature of care work in the space of early childhood. Early childhood workforces are female-dominant [45] and approaches to early intervention are often heavily associated with mothers [46]. In this sense, privileging women voices and providing space for them to frame key issues through PAR was potentially key to unveiling our findings relating to the socio-political and socio-economic environments which prevent access to childhood health and services. However, it may mean a missed opportunity for capturing a more gender-diverse perspective, and engaging a more diverse pool of practitioners to engage in this process and realise their agency in creating change [47]. Alternatively, or perhaps additionally, this observation may in and of itself also point towards limitations in the ABSS programmes offered and challenge the inclusivity of fathers or gender-diverse parents within these.

Finally, it is helpful to contextualise PAR as one component within a larger evaluation of ABSS, which included greater diversity of methods and participants. Through questionnaires, surveys and focus groups, data has been collated from programme managers, other staff as well as parents both engaged, and not engaged with ABSS services. This is supplemented by multiple sets of quantitative data collected and analysed in relation to specific child health and development outcomes. A PAR approach, that enables and centres reflexivity in the practitioners engaging with the process of analysis, whilst employing intersectionality as an epistemological starting point could be a vital process to ensure a liberatory agenda is garnered towards [44, 48]. This highlights the key agential role and position the health and social care worker occupies in elucidating marginalised experiences. PAR offers the possibility to develop this further: in particular, to explore the role of the healthcare worker in building analysis and action in the space between their role and the wider system they, and we all, are located in.

## Conclusion

This study illustrates how operationalising PAR in an evaluation of a place-based early years public health programme can help generate nuanced and detailed insights on the major barriers for the community in accessing good child and maternal health. Adopting this with health practitioners delivering services brought them into a generative space that was created to identify problems, consider solutions and produce an action agenda, which was evidenced through knowledge, knowledge-in-action and transformation. We consider that utilising PAR was especially powerful in framing critical evaluation points not just in terms of process (for example, issues with staffing), logistics (for example, the limitation of ABSS to only selected wards in Southend) or resource (for example, lack of interpreters) that perhaps are the focus in more traditional evaluation methods, but broader socio-economic and socio-political factors. This leads us to recommend PAR as an approach that could also be embedded for service design and delivery in future.

Ultimately, though advancing access to specific services and assets and benefiting parts of the community, our research indicates that the ABSS programme may have fell short in effectively enhancing access to health services for all, due to the unsurmountable influence of broader socio-economic and socio-political determinants – such as the cost of living, housing, ethnicity-based exclusion and deprivation. Future public health EYIs would benefit from taking an intersectional, multi-systems approach to ensure those on the margins of society can benefit. The equity dimension of EYIs should be evaluated through a range of diverse methods including embedding PAR from the onset, and for final evaluations. Consideration of PAR tools to sustain consciousness-raising and transformation of and among participants and community agendas is worthwhile. In the context of intersectional work with marginalised groups, that needs to account for historical barriers and on-going unequal power dynamics, PAR has much to offer through long-term, sustained engagement [44, 48].

## Data Availability

The datasets generated and/or analysed during the current study are not publicly available due their procurement for a commissioned evaluation only and are not for wider use.

## Abbreviations

PAR: participatory action research
ABSS: A Better Start Southend
